# Gene Promoter Evolution Targets the Center of the Human Protein Interaction Network

**DOI:** 10.1371/journal.pone.0011476

**Published:** 2010-07-08

**Authors:** Jordi Planas, Josep M. Serrat

**Affiliations:** Bioinformatics and Medical Statistics Group, Department of Systems Biology, Universitat de Vic, Vic, Spain; University of Texas Arlington, United States of America

## Abstract

Assessing the contribution of promoters and coding sequences to gene evolution is an important step toward discovering the major genetic determinants of human evolution. Many specific examples have revealed the evolutionary importance of *cis*-regulatory regions. However, the relative contribution of regulatory and coding regions to the evolutionary process and whether systemic factors differentially influence their evolution remains unclear. To address these questions, we carried out an analysis at the genome scale to identify signatures of positive selection in human proximal promoters. Next, we examined whether genes with positively selected promoters (*Prom^+^* genes) show systemic differences with respect to a set of genes with positively selected protein-coding regions (*Cod^+^* genes). We found that the number of genes in each set was not significantly different (8.1% and 8.5%, respectively). Furthermore, a functional analysis showed that, in both cases, positive selection affects almost all biological processes and only a few genes of each group are located in enriched categories, indicating that promoters and coding regions are not evolutionarily specialized with respect to gene function. On the other hand, we show that the topology of the human protein network has a different influence on the molecular evolution of proximal promoters and coding regions. Notably, *Prom^+^* genes have an unexpectedly high centrality when compared with a reference distribution (P = 0.008, for Eigenvalue centrality). Moreover, the frequency of *Prom^+^* genes increases from the periphery to the center of the protein network (P = 0.02, for the logistic regression coefficient). This means that gene centrality does not constrain the evolution of proximal promoters, unlike the case with coding regions, and further indicates that the evolution of proximal promoters is more efficient in the center of the protein network than in the periphery. These results show that proximal promoters have had a systemic contribution to human evolution by increasing the participation of central genes in the evolutionary process.

## Introduction

Early observations of low levels of protein divergence between humans and chimpanzees have suggested that most evolutionary changes in the human lineage have occurred at the regulation level [1]. Since these observations were made, a number of studies have established that some *cis*-regulatory regions play a key role in the evolution of the phenotype. For instance, *cis*-regulatory elements for human genes related to immune responses, dietary changes and behavior and cognition show signatures of molecular evolution [2], [3]. However, the impact of evolution on *cis*-regulatory regions at a genome-wide scale has not been undertaken until recently. Signatures of positive selection in promoter regions are widespread all over the genome, affecting about one tenth of the genes [4]. From genome-wide studies, it has been concluded that the promoters of genes related to neural- and nutrition-related processes show signatures of positive selection [4], a tendency they share with positively selected proteins [5]. In a step forward, the finding that there is a high probability of positive selection in *cis*-regulatory regions near genes expressed in the fetal brain [6] highlights the importance of regulatory regions in human evolution.

Genome-wide analysis also opens the door to a more systemic approach from which, for instance, one can infer general evolutionary principles. In regulatory sequences, as in transcribed regions, the success of evolutionary changes depends on successful changes at the molecular and system level. The products of genes interact in a concerted manner to accomplish their functions; thus, their evolution is not independent of the set of molecular interactions occurring in the organism at a given time or place. In the past few years systems biology has analyzed the structures of the protein interaction networks for several species. This has enabled to study the evolution of human proteins in a network context. It has been established that some features of network topology influence the rate of protein evolution. For instance, proteins with many connections are conserved to a greater extent than proteins with few connections [7], [8], [9], [10]. Although the influence of the network topology on the evolution of regulatory sequences has not been investigated, there are data to indicate that some effects may exist. In this regard, it has been reported that the expression levels of interacting proteins are evolutionarily coupled [11].

Given the evolutionary importance of regulatory regions, we believe that the following basic question merits further exploration: is there any critical systemic difference between the contributions of regulatory and coding regions to the evolution of the human lineage?

In order to address this question, we studied genes that have accumulated enough mutations to be considered as being under positive selection pressure. To identify these genes, we carried out a genome-wide evolutionary analysis of human proximal promoters; this is the ∼1 kb region upstream of the transcription start site that hosts the greatest concentration of nucleotides belonging to transcription factor binding sites [12], [13], [14]. We obtained the data on the molecular evolution of coding regions of proteins from a study in which chimp and macaque served as the reference species [15], as in our analysis.

To obtain insight into the biological significance of the evolutionary changes, we looked for differential trends between positively selected proximal promoters and proteins. There are at least three general approaches. First, the number of genes with positive selection in the promoter could differ from the number of positively selected proteins. Second, the question of whether some biological processes have been specifically targeted through promoter evolution can be revisited with the new data. Third, a comparison of network topology features for positively selected promoters and proteins could provide additional information. Protein-protein interaction networks are useful to obtain information about the structural determinants of promoter evolution. Mutations in the promoter region may influence the rate of transcription and consequently may affect the concentration of a given protein in the cell. In most cases, a change in concentration may give rise to changes in the kinetics of the reactions in which the protein is involved. The effect of these changes on the cell will depend on the position of a protein in the network as well as on the particular function of the involved protein. Thus, for a particular gene, mutations in the promoter region may be negatively selected or may be an opportunity for positive selection depending on how changes in the concentration of its coded protein affect the functioning of the cell. Further aspects of the structural determinants of the evolution of promoters will likely be revealed when comprehensive protein-DNA interaction networks are available. Our results show that (i) there are no differences between the number of genes with positive selection in the proximal promoter and the number of proteins with positive selection, (ii) positive selection is widespread over all biological functions including those affecting critical processes such as cell proliferation and differentiation, cell cycle and mRNA transcription, and (iii) unlike proteins, genes with positively selected proximal promoters are more central than expected in the human protein interaction network, which might be an indication of their relevant role in human evolution.

## Results

### Analysis of the molecular evolution of proximal promoters

We aligned 17067 human proximal promoters from protein coding genes with the orthologous sequences of chimp and macaque. Among the various algorithms for aligning multiple sequences, we selected the alignment approach implemented in the PRANK program [16]. PRANK, unlike most algorithms, resolves alignments by taking into account the phylogenetic coherence of the deletions and insertions that occur during the evolutionary process [17]. We consider this a major improvement, in that it could help to decrease the number of false positives and false negatives in the subsequent analysis of molecular evolution. At the end of the process, after a number of data filtering steps (see Methods), we retained 5892 alignments for further analysis. To look for signatures of positive selection in promoters we used the method developed by Haygood *et al.* [4]. This method is based on a comparison between two single-nucleotide substitution models that are sensitive to positive selection rather than to the relaxation of negative selection. The codes of the genes included in this study, the step in the process in which genes were filtered out and the codes of the genes that eventually passed the evolutionary analysis are shown in Table S1.

Considering our data along with Berglund's data [15], we found that the number of positively selected promoters (477 of 5892 analyzed genes, 8.1%) and proteins (406 of 4779 analyzed genes, 8.5%) is not significantly different (*P* = 0.5, two-tailed Fisher's exact test). In previous studies that used similar methodologies, Haygood *et al*. [4] found 457 genes showing positive selection at the 5 kb region upstream of the TSS in a set of 4959 analyzed genes (9.2%), and Clark *et al.* [5] found 524 genes with positive selection in the coding region among 6094 genes analyzed (8.6%).

A related question is the possibility of a certain degree of coevolution of promoters and coding regions. There are 1973 genes for which we know the evolutionary status of both the promoter (178 with positive selection) and the coding region (172 with positive selection). Thus, assuming independence, there should be 15 genes with positive selection in both the promoter and the coding region, a value that is not significantly different from the 18 genes that we observed (*P* = 0.7, two-tailed Fisher's exact test).

Hereafter, the set of genes with positively selected proximal promoters and Berglund's set of genes with positively selected coding regions will be referred to as *Prom^+^* genes and *Cod* ^+^ genes, respectively.

### Functional analysis of genes with signatures of positive evolution

To gain insight into the functional landscape of genes with signatures of positive selection, we generated a custom slim containing 38 terms of the PANTHER database [18], basically the top parent terms of the ontology. We performed a hypergeometric test to identify functional categories where the number of genes with positive selection is different from what would be expected in a random sample [19].

First, we analyzed *Prom^+^* genes and *Cod^+^* genes separately (Table 1, Table S2). At first glance, the functional categories that are enriched/impoverished in the two groups are not the same. Enriched categories might indicate that certain processes are a target of evolution, meaning that the evolution of a lineage is characterized by changes in one or several groups of genes that are involved in the same biological process. Conversely, impoverishment would suggest either low evolutionary interest or specific constraints on introducing molecular changes in the genes related to a biological process. However, functional analysis could be performed from a broader perspective, not only considering enriched/impoverished categories but also taking into account the number of genes falling into these categories. From this point of view, 21% of *Prom^+^* genes fall into one of the two enriched categories (Protein metabolism and Metabolism), while just 6% of *Cod^+^* genes lie in one of the three enriched categories (Muscle contraction, Sensory perception and Phosphate metabolism). Impoverished categories (Cell communication, mRNA transcription and Signal transduction) contain 16% of *Prom^+^* genes, while there are no impoverished categories with *Cod^+^* genes. Thus, these two set of genes are not globally different from a functional perspective.

**Table 1:** Functional analysis of the *Prom^+^* genes, *Cod^+^* genes and genes showing signatures of positive selection either in the proximal promoter or the coding region.

	*Prom^+^* genes		*Cod^+^* genes		Positive genes	
	PANTHER Ontology terms	n	PANTHER Ontology terms	n	PANTHER Ontology terms	n
**Enrichment**	Protein metabolism	83	Muscle contraction	8	Phosphate metabolism*	12
	Other Metabolism	22	Sensory perception	11	Protein metabolism	140
			Phosphate metabolism	5	Carbohydrate metabolism	34
**Impoverishment**	Cell communication*	13			m-RNA transcription	55
	m-RNA transcription	23			Signal transduction	119
	Signal transduction	54			Cell communication	39

Categories with significantly more (enrichment) or less (impoverishment) genes than expected (P<0.05, Hypergeometric test).

*Prom^+^* genes, n = 477.

C*od^+^* genes, n = 406.

Genes showing signatures of positive selection either in the proximal promoter or the coding region (Positive genes), n = 871.

*P<0.01.

Next, we reasoned that specific selective pressure demands could be met by fixing changes in promoters and coding regions alike. Thus, we repeated the analysis without discriminating between promoters and coding regions, considering all genes that have signatures of positive selection either on the promoter or on the coding region (Table 1, Table S2). When *Prom^+^* genes and *Cod^+^* genes are analyzed as a single set, the patterns of enrichment and impoverishment are similar to those observed for *Prom^+^* genes. Moreover, 19% of the genes are in enriched categories and 19% are in impoverished categories.

Finally, we considered that genes showing signatures of positive selection in both the promoter and the coding regions are a subset of special interest because they might point to evolutionary hotspots. Because some of these genes are not yet classified in the PANTHER database, we used Gene Ontology [20] and UNIPROT annotations [21] in order to assign each of these 18 genes to one parent PANTHER category at least. Only one gene, ZC3H18, has no functional annotation. In Table 2 we show the genes within categories containing more than two genes. With regard to functional analysis (Table S3), Cell proliferation and differentiation (*P* = 0.01), Developmental processes (*P* = 0.01), and Cell cycle (*P* = 0.02) not only show a significant enrichment but also contain 14 of the 17 genes with positive selection in both the promoter and the coding region.

**Table 2:** Functional classification of the genes showing signatures of positive selection both in the proximal promoter and the coding region.

Biological process*	Gene symbol
Cell proliferation and differentiatiom	ANP32B, DSTYK, PIK3R2, NCAN, GEMIN4, DKK2, CCDC134
Development	NEIL3, CHORDC1, DKK2, CCDC65, NCAN, UBP1
Nucleoside, nucleotide and nucleic acid metabolism	NEIL3, EME1, TRUB1, SFRS14, UBP1
Signal transduction	DSTYK, PIK3R2, DKK2, CCDC134
Protein metabolism	ANP32B, CHORDC1, PSMC3, DSTYK
Cell Cycle	EME1, ANP32B, SMEK2, PSMC3

*PANTHER Ontology terms containing more than two genes with signals of positive selection both in the promoter and the coding region.

### Comparison between the centrality of the positively selected proximal promoters and proteins

Protein interaction networks are non-random and the number of interacting partners of their nodes is not normally distributed and follows a heavy-tail distribution. In this kind of network, the most central nodes have an important role in the topology of the network. In searching for systemic determinants of promoter evolution, we examined the relationship between the centrality of genes in the protein interaction network and positive selection. To this end, we used the IntAct database as a reference for the human protein interaction network [22]. IntAct is a curated database that, as found with other initiatives, represents only a fraction of the human protein interactome. As a consequence, only a fraction of *Prom^+^* genes and *Cod^+^* genes are present in the IntAct network: n = 188 and n = 152 genes, respectively (for the sake of simplicity we will also refer to these subsets of genes as *Prom^+^* genes and *Cod^+^* genes). In order to perform the analysis with as many genes as possible, we assumed a certain degree of contamination in the sense that some genes might have both positively selected promoter and coding regions. Nevertheless, we have estimated that this occurs in only 0.8% of genes.

**Figure 1:**
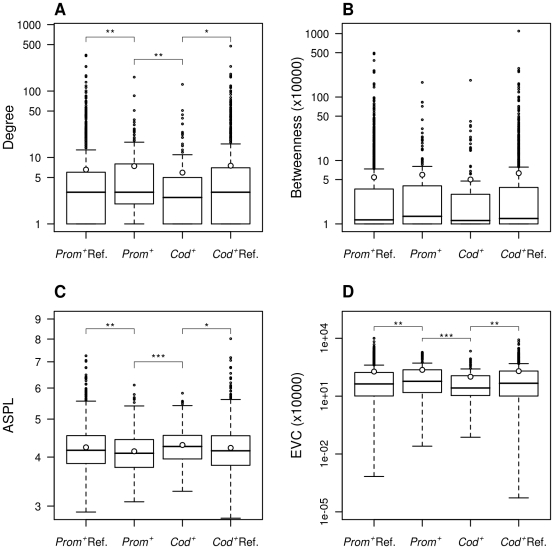
Distributions of the centrality parameters of the IntAct network proteins. **A.** Distribution of the degree. The degree of a node, also known as connectivity, is the number of its interacting partners. It is a local measure of centrality, which means that it is not affected by the topology of other regions in the network. **B.** Distribution of the betweenness. Betweenness centrality is a parameter that is roughly defined as the number of shortest paths between pairs of nodes in the network that pass through a given node and is interpreted as a measure of the importance of a node for the flow of information through the network. As the betweenness distribution contains zeros, we have added 1 unit to the betweenness values to be able to plot the distribution in log scale. **C.** Distribution of the ASPL. The ASPL value of a node is the average shortest path length between the node and all other nodes in the network and can be interpreted as a measure of geometrical centrality. Notice that the more central a node, the smaller its ASPL. **D.** Distribution of the Eigenvalue centrality (EVC). The EVC of a node is its associated score in the eigenvector of the largest eigenvalue of the adjacency matrix; in a protein network with unweighted edges, nodes with high EVC are those that are connected to many nodes, which are, in turn, connected to many other nodes and so on. *Prom^+^*: genes with positively selected proximal promoters (n = 188); *Cod^+^*: genes with positively selected coding regions (n = 152); *Prom^+^* Ref.: the reference set for *Prom^+^* (n = 2219); *Cod^+^* Ref.: the reference set for *Cod^+^* (n = 1811). The open circle shows the mean of the distributions. *P<0.05, **P<0.01 and ***P<0.001.

Centrality parameters, among the parameters used to characterize the nodes of a network, measure the importance of each node [23]. Degree centrality, average shortest path length (ASPL), betweenness and eigenvector centrality (EVC), though correlated to some extent, convey different notions of centrality (see legend Figure 1). First, we studied the distribution of these parameters in the *Prom^+^* and *Cod* ^+^ genes (Table S4) and then we examined whether the number of positively selected genes was associated with centrality.

The results indicate that the centrality of *Cod^+^* genes is lower than that of a reference set (n = 1811), as clearly shown in the case of degree centrality (one-tailed Wilcoxon-Mann-Whitney test (WMW), *P* = 0.037), ASPL (WMW, *P* = 0.01), and EVC (WMW, *P* = 0.006). This trend is in agreement with previously reported observations [10] [1]. To our surprise, *Prom^+^* genes showed an inverse trend; proximal promoters were found to be more central than expected (reference set, n = 2219) in the case of degree centrality (WMW, *P* = 0.009), ASPL (WMW, *P* = 0.004), and EVC (WMW, *P* = 0.008) (Figure 1, Table S5, Table S6).

**Figure 2:**
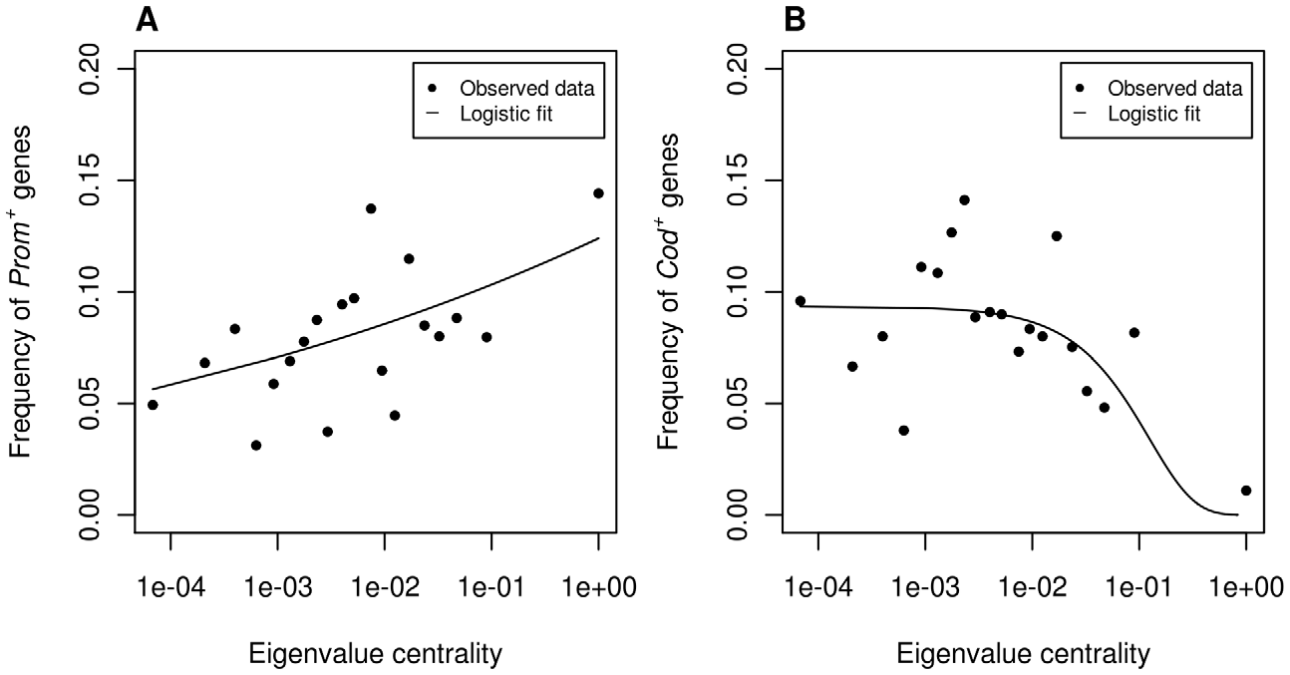
Association between the frequency of positively selected genes and centrality. **A.** Logistic regression between the frequency of *Prom^+^* genes and Eigenvalue centrality (EVC). **B.** Logistic regression between the frequency of *Cod^+^* genes and EVC. In both panels, the X axis values correspond to the upper interval quantile of EVC in log coordinates.

In Figure 2, we graphically show the relationship between centrality (EVC) and the frequency of *Prom^+^* genes and *Cod^+^* genes. Data were fitted with a logistic regression using a binary outcome. To each EVC value we associated a 1 value if the corresponding gene was positively selected and a 0 value otherwise. The EVC values of *Prom^+^* genes were log transformed before fitting. Data were fitted using the *glm* function of the R package [24]. To graphically display the observed data, we broke the EVC data (a continuous variable) into 20 categories, corresponding to quantile intervals, and we plotted the frequency of positive genes against the upper value of each centrality interval.

Regression coefficients were 0.09 (*P* = 0.02) and - 8.6 (*P* = 0.02) for *Prom^+^* genes and *Cod^+^* genes, respectively. Although these two values are not quantitatively comparable due to the log transformation in *Prom^+^* genes, the opposite sign of the regression coefficients indicates an inverse trend in the corresponding relationships: the frequency of *Prom^+^* genes increases with centrality and the frequency of *Cod^+^* genes decreases. In the case of *Cod^+^* genes, we see from the fitted data that the frequency of *Cod^+^* genes is rather constant at the periphery of the protein network and then decreases rapidly at the center. The results show that the frequency of *Prom^+^* genes is greater in the center of the protein network than in the periphery.

### Proximal promoter evolution is not constrained by the level of gene expression

Although the causal links are not conclusive [25], [26], some evidences indicate that one of the chief factors constraining protein evolution is expression level [27], [28], [29]. Taking into account the determinant role of the promoter region in the expression of a gene, we studied the relationship between positive selection and gene expression. We examined whether the expression levels for *Prom^+^* genes and *Cod^+^* genes were significantly different from a random reference set. As the sets were large enough, for this analysis we preferred to use genes for which we knew the evolutionary status of both the promoter and the coding region. Using the highest expression value per tissue and per gene (DATA1) and using the average of the expression values equal or greater than the median (DATA2) for each gene and set of tissues, we observed that the expression values for the Cod^+^ genes were lower than expected (n_1_ = 120; reference set, n_2_ = 1522; WMW, *P* = 0.03, for DATA1; WMW, *P* = 0.03, for DATA2), which is in agreement with observations reported elsewhere [30]. In contrast, we observed that the expression values of the Prom^+^ genes were no different from the reference set (n_1_ = 132; reference set, n_2_ = 1522; WMW, *P* = 0.08, for DATA1; WMW, *P* = 0.38, for DATA2) (Table S7). These results indicate that expression is not a constraint on proximal promoter evolution.

### Correlation between the level of gene expression and gene centrality

In order to investigate the unexpected level of expression encountered in the *Prom^+^* genes, we examined the relationship between expression and centrality in the genes (*i.e.* their coded proteins) present in the IntAct network. Using Kendall's non-parametric method, we observed a slight but significant correlation between the level of expression and the centrality parameters under study (Table 3). Because the correlations were small, we questioned whether the group of genes analyzed in our study (a sample of the IntAct network) retain a significant correlation. We discovered that *Prom^+^* genes exhibit a significant correlation between the level of expression and ASPL or EVC. On the other hand, the expression levels of *Cod^+^* genes were not correlated with any of the centrality parameters under study (Table 3).

**Table 3:** Correlation between centrality and level of expression.

	DATA1^1^						DATA2^2^					
	IntAct proteins		*Prom^+^* genes		*Cod^+^* genes		IntAct proteins		*Prom^+^* genes		*Cod^+^* genes	
	*tau*	p-value	*tau*	p-value	*tau*	p-value	*tau*	p-value	*tau*	p-value	*tau*	p-value
**Degree**	0.052	**<10^−6^**	0.041	0.5	0.035	0.6	0.048	**<10^−7^**	0.041	0.5	0.040	0.5
**Betweeneness**	0.058	**<10^−10^**	0.068	0.2	0.043	0.5	0.056	**<10^−9^**	0.073	0.2	0.051	0.4
**ASPL**	−0.071	**<10^−14^**	−0.14	**0.006**	−0.058	0.3	−0.071	**<10^−15^**	−0.15	**0.005**	−0.034	0.6
**EVC**	0.071	**<10^−16^**	0.14	**0.008**	0.062	0.3	0.071	**<10^−15^**	0.14	**0.008**	0.042	0.5

Intact proteins, n = 6099.

*Prom^+^* genes, n = 164.

*Cod^+^* genes, n = 142.

1 Using per each gene the highest expression value encountered in the set of tissues included in the E-GEOD-803 experiment.

2 Using per each gene the average of the expression values equal or greater than the median of the set of tissues included in the E-GEOD-803 experiment.

### Functional analysis of central genes

As centrality seems to be an important parameter for evolution, acting either directly or as a reporter of other underlying variables, we decided to perform a functional analysis of the most central genes. We considered a 20 gene set containing the top ten central *Prom^+^* genes together with the top ten central *Cod^+^* genes and determined what kind of biological processes they were involved in (Table 4). We then performed a functional analysis (Table S8) and we found enrichment in Cell proliferation and differentiation (*P*<0.001), Protein metabolism (*P* = 0.004), Cell cycle (*P* = 0.002), Signal transduction (*P* = 0.01), Intracellular protein trafficking (*P* = 0.02), and mRNA transcription (*P* = 0.02). Then, we questioned whether these categories show enrichment because of a distinct feature among positively selected central genes or because central genes tend to be enriched in these categories. In order to address this question, we repeated the functional analysis using as a reference set the IntAct proteins having an EVC value higher than the minimum EVC value observed for the positively selected central genes, as defined above (Table S9). In this second analysis, we can see that the number of positively selected genes involved in Cell proliferation and differentiation (*P*<0.001), Signal transduction (*P* = 0.005), and mRNA transcription (*P* = 0.007) is higher than expected. This enrichment indicates that in the center of the network, these biological processes are a target of natural selection. The remarkable aspect of the enrichment in these three categories is that 60% of the more central positively selected genes fall at least into one of them.

**Table 4:** Functional classification of the positively selected central genes.

Biological process*	Gene symbol
Cell proliferation and differentiatiom	NCK1, TRAF1, NFKBIA, NKX2-1, NEK6, CCDC85b, SSR1, SMNDC1
Signal transduction	NCK1, MAP3K8, RANBP1, TRAF1, SSR1, NEK6, DAG1, NFKBIA
Protein metabolism	MAP3K8, RANBP1, PSMC4, SSR1, NEK6, PFDN1, CANX, LRPPRC
Transcription and m-RNA processing	LRPPRC, SMNDC1, NUDT21, NKX2-1, DDX5, CCDC85B, NFKBIA
Nucleoside, nucleotide and nucleic acid metabolism	CTPS, NFKBIA, DDX5, SMNDC1, NKX2-1, NUDT21
Cell cycle	MAP3K8, PSMC4, PFDN1, NEK6, SSR1
Intracellular protein traffic	RANBP1, NFKBIA, SSR1, CANX
Development	NCK1, VIM, NKX2-1

Positively selected central genes: the set of twenty genes containing the top ten central *Prom^+^* genes and the top ten central *Cod^+^* genes.

*PANTHER Ontology terms containing more than two genes with signals of positive selection.

## Discussion

We present a genome-wide analysis looking for signatures of positive selection in a very critical region for gene regulation, the 1 kb region upstream of the TSSs for the human genes. This analysis is important to the study of gene evolution and regulation because *cis*-regulatory sequences are the main determinants for gene expression [31] and because the proximal promoter contains the greatest concentration of nucleotides that constitute transcription factor binding sites [32], [13], [14], which are responsible for controlling the level of expression [12], [33]. Therefore, our results cannot be interpreted as a general feature of all *cis*-regulatory regions, but as a specific feature of proximal promoters. It has been claimed that in some circumstances, if not most cases, regulatory regions are more amenable to evolve than coding regions [3]. Mutations in regulatory regions are usually co-dominant [34], [35] and hence are readily accessible to natural selection in contrast with the recessive nature of mutations in coding regions. Moreover, mutations in regulatory regions are able to produce a continuous spectrum of phenotypic changes [36], which may make it easier to meet selective pressure demands. One consequence of this solid rationale is that the number of genes with positively selected promoters should be higher than the number of genes with positive selection in their coding regions. Here, we show that this is not the case. Therefore, we conclude that, from a quantitative point of view, proximal promoters and proteins make an equivalent contribution to the evolution of the human lineage. This conclusion is based on an analysis of positive selection performed at the proximal promoter level, taking into account the number of human specific substitutions in this region with respect to a neutral intronic region. We believe that a different conclusion might arise when an analysis of positive selection can be performed at the level of transcription factor binding sites, an investigation that is not possible with currently available data. On the other hand, the detection of positive selection in coding regions using an evolutionary model based on *d*_N_/*d*_S_ might suffer from several sources of bias [15], [37]. It may well be that future methods adjusting for these biases change the number of positively selected coding regions, invalidating this first conclusion. In general, the effect of these biases results in a number of false positives increasing the noise of the gene set under analysis, but it does not necessarily affect the rest of the analysis in this study from a qualitative point of view.

Next, we performed a functional analysis with the aim of answering two main questions: (i) Is there a functional specialization of promoters and proteins so that genes related to some biological processes evolve mainly through changes in promoters while genes related to other biological processes evolve through changes in protein-coding regions? (ii) Is there any biological process that has accumulated critical evolutionary changes? Concerning the first question, we saw that 81% of *Prom^+^* genes and 94% of *Cod^+^* genes are in non enriched categories. Moreover, even if a category is enriched in *Prom^+^* genes, there is a large contribution from *Cod^+^* genes; for example, in Protein metabolism we found 83 *Prom^+^* genes and 59 *Cod^+^* genes. Therefore, we show that positive selection is affecting almost all biological processes, indicating that proximal promoters and coding regions are not evolutionarily specialized with respect to gene function. Previous works have reported a high probability of positive selection associated with promoters of genes involved in developmental processes [4], [6], [38]. These results are important because they show that evolutionary changes in developmental genes may have been driven by regulatory sequences. In contrast, our results do not reveal the same tendency. This could be due to the fact that in developmental genes the distal part of the promoter is the one that has played the key evolutionary role in fine-tuning gene expression, as suggested previously [14]. But the fact that the method that we have used to detect enrichment in functional categories is different from those used in other studies might also account for the results. For instance, Hoffman and Birney found high probability of positive selection associated with proximal promoters of developmental genes [39], which could indicate that methodology also influences functional analyses. However, though enrichment in some categories might provide valuable biological information, as for example that some functions have evolve mainly through changes in regulatory regions, the main conclusion we can draw from the functional analysis is that the evolution of a species is a complex process, involving many changes in promoters and coding regions distributed over the spectrum of biological processes.

On the other hand, when we analyzed genes of special interest either because they show signatures of positive selection in the promoter and coding region or because they are the most central of *Prom^+^* and *Cod^+^* genes, we observed that most of these genes belong to enriched categories (Cell proliferation and differentiation, Development, Cell cycle, Signal transduction and mRNA transcription). The fundamental role that we can bestow to these categories suggests that human evolution may have been driven by genetic changes that can entail great phenotypic changes.

We have shown that either from a quantitative point of view or from a functional perspective there are no systemic differences influencing the evolution of proximal promoters and proteins. However, a systemic determinant of proximal promoter evolution does exist. We show that proximal promoter evolution targets the center of the human protein network. We found that the centrality of *Prom^+^* genes is higher than a random reference and that the frequency of these genes increases from the periphery to the center of the network. Previous reports on topological factors influencing molecular evolution have always described negative correlations [40]. Here, we describe a positive effect between a topological parameter and the evolution of a gene region.

The major systemic determinants of protein evolution - level of expression [29] and structural constraints [9], [41], [42], [43]- are fairly well understood. The sequence evolution rate for highly expressed proteins is constrained at the translational level (the translational hypothesis). It is thought that the accumulation of misfolded structures for highly expressed proteins can severely damage the cell, so well optimized sequences leading to a high ratio of correct/incorrect three-dimensional structures are strongly preserved. As for structural constraints, changes in the sequence of a protein that interacts with many other proteins, a hub protein, are unlikely accommodated to preserve all partner interactions, resulting in deleterious effects for the cell. In consequence, degree and rate of sequence evolution correlate negatively [7], [10], [44]. The influence of these two factors on the rate of protein sequence evolution is of comparable magnitude [45].

Our results indicate that the evolution of proximal promoters is not affected by the same factors that constrain protein evolution. On the one hand, *Prom^+^* genes display a higher level of expression than a random reference. Furthermore, we found a positive correlation between the centrality of *Prom^+^* genes and their level of expression. Since in the entire protein network gene centrality is positively correlated with gene expression, *Prom^+^* genes follow the main trend of the network. In contrast, we observe a constraint on the expression level for *Cod^+^* genes. *Cod^+^* genes display a lower than expected level of expression, and their centrality is not correlated with their level of expression. This lack of correlation might be because the global correlation weakens at the periphery of the network, and as *Cod* ^+^ genes are less central than expected the correlation in this subset of genes is lost. On the other hand, the relatively high centrality of *Prom^+^* genes and the positive association between the frequency of *Prom^+^* genes and centrality might reveal that changes in the spatiotemporal rewiring of the network and in the expression of central genes, due to changes in promoters, might be better tolerated than permanent changes in the connectivity of proteins, which is often the result of a mutation in a coding region. This is in agreement with previous observations suggesting that the evolution of transcription is unconstrained [46]. Furthermore, these results indicate that the evolution of proximal promoters is more efficient at the center of the protein interaction network. It is likely that the more central in the network a gene is (i.e. its coded protein), the greater the phenotypic changes that a mutation in the promoter region can produce. In a recent work by Haygood et al. [38], the authors show that positive selection in promoters is associated with the evolution of complex phenotypes, which is the expected result when the involved genes are central in the protein interaction network. We hypothesize that, in a wide spectrum of selective pressures, this sort of changes confer greater fitness to the organism than the type of changes produced by mutations in peripheral promoters.

Central genes are biologically important in the sense that most of them are pleiotropic (involved in multiple phenotypes) and often non dispensable [42], *i.e.,* the organism is not viable without these genes. Consequently, we suggest that proximal promoters have contributed to human evolution by increasing participation of central genes in the evolutionary process. Thus, assuming that mutations in central genes have a high phenotypic impact, our results indicate that some large phenotypic changes, driven by promoter evolution, may have boosted the evolution of the human lineage. An extended analysis of network structure, together with the inclusion of other genomes over a wide range of clades, will confirm whether this is a specific trend of human evolution and will shed light on the causes underlying the high centrality of positively selected proximal promoters.

## Methods

### Molecular evolution of human proximal promoters

#### Human proximal promoters and intronic region sequences

All human genomic coordinates were obtained with BioMart [47] from data set NCBI36 of Ensembl 49 [48]. Sequences spanning from -1100 to +150 bases from the start site of all human protein coding genes were downloaded from the Ensembl 49 database. The corresponding hard masked sequences were also downloaded. Intronic regions were defined as those parts of the introns that overlap neither with exons of alternate transcripts nor with exons of overlapping genes. The coordinates of intronic regions were obtained by merging all overlapping exons and keeping the remaining non coding regions inside the genes. Next, for further analysis, we discarded intronic sequences shorter than 600 kb and larger than 6200 kb. Intronic regions and their corresponding hard masked sequences were downloaded from Ensembl.

#### Orthologous sequences

The coordinates of orthologous human, chimp and macaque genes were obtained from the Ensembl 49 database using Biomart. Only genes that were present in the three species and annotated in a main chromosome were retained for further analysis. To compile the set of orthologous sequences, the full set of chromosomes of *Pan troglodytes* (PanTro2 assembly) and *Macaca mulatta* (MacRhe2 assembly) were downloaded. Chromosome sequences were locally indexed as Blast databases. Using the masked sequences of the human promoters and intronic regions as queries, standalone Blast searches were performed against the chromosome where the chimp and macaque gene orthologs are located according to the Ensembl annotation. Default Blast parameters were used except for the word length (-W) and expect (-E): we used -W 28 and -W 14 for chimp and macaque searches and -E 0 for both. When Blast failed to find a hit, a second round with a shorter word length was performed: -W 14 for chimp and -W 7 for macaque. For each search, the hit with the highest score was selected. Then, as the Blast output returns the coordinates of a partial alignment between the query and target sequences, the matched sequence was expanded to achieve the length of the query sequence. Those promoter sequences whose TSS coordinate in the chimp or macaque lay more than 10 kb away from the Ensembl annotated TSS in the corresponding chain were excluded from further analysis.

#### Alignment of orthologous sequences

Orthologous sequences were aligned by running PRANK [16] with the -F option. In order to eliminate questionable results, alignments were tracked with a 50-base sliding window. Any alignment having one or more windows with more than 12 differences, excluding indels, between the human and chimpanzee sequences or 17 differences between the human and macaque sequences was rejected. Alignments showing more than 10% site gaps were also discarded from further analysis (Table S1).

#### Evolutionary analysis

Evolutionary analysis of the human proximal promoters was performed using the method and tools developed by Haygood *et al*. [4] Roughly, this method uses the alignment of the promoter and the alignment of its flanking intronic regions to compare two evolutionary models. It can be considered that the sequence of a promoter has experienced positive selection if the p-value associated with the likelihood ratio test used to compare the two models is <0.05. To build the intronic references, 100 bases at the ends of each intronic sequence were discarded before merging all intronic regions within a region 10 kb upstream of the human TSS and 10 kb downstream of the end of the terminal exon of the gene. The first intron of each gene was excluded from this process. To ensure the neutrality of the intronic reference, alignments with a ratio of human specific substitutions outside the interval 0.0053±2 s.d. (s.d.* = 0.0022*) were rejected (Figure S1). The segment of the promoter alignment corresponding to the coordinates of the human proximal promoter (1 kb) and the corresponding intronic reference were used to fit the parameters of the models (Table S1).

### Functional analysis

We downloaded PANTHER classifications from HMM Library Version 6.1 (ftp://ftp.pantherdb.org) [18]. We built a slim containing all parent categories together with some children categories that we find especially interesting (Table S2). We matched our genes and Berglund's genes [15] with Ensembl identifiers. For this reason, Entrez identifiers in the PANTHER database were converted to Ensembl identifiers. For each PANTHER category, we computed the probability of overrepresentation and underrepresentation of genes with positively selected promoters, genes with positively selected coding regions and genes with positive selection in either the promoter or the coding regions. We used the R functions *phyper* and *dhyper* as described elsewhere [19].

### Computation of the parameters of the IntAct protein interaction network

We downloaded the full IntAct protein network database (September, 2009) to select entries corresponding to pairs of human protein interactions [22]. The IntAct database contains information on approximately half of the human genes. Consequently, only 188 proteins encoded by genes with positively selected promoters and 152 proteins corresponding to positively selected coding regions were represented in the human protein network. Degree, betweenness, average shortest path length (ASPL) and eigenvector centrality were computed based on the largest component of the network using R package Igraph (Table S4). We have examined whether the bias introduced by weak to strong mutations (AT to GC) influences the centrality results (Text S1).

### Expression level of the human genes

We obtained the expression level of the human genes in normal tissues from the ArrayExpress database, E-GEOD-803 processed file, GEO accession GSE803 [49]. Where the cross reference was available, Affimetrix composite element references (GeneChip Human Genome U95A-E) were translated to gene Ensembl codes. We retained values belonging only to normal tissues and claimed as significant (“Present”) by the authors. As our interest was in the highest levels of expression of a gene, we assigned the highest value encountered to each gene in the set of tissues showing expression for that gene (DATA1) and also the average of the expression values equal to or greater than the median of the distribution in each tissue set (DATA2).

## Supporting Information

Figure S1 Distribution of the human specific substitutions per site in the intronic regions. The average human specific substitution ratio of the alignments of the intronic regions was 0.0053 and the standard deviation (s.d.) was 0.00219.
(3.51 MB DOC)

Table S1 Data of the alignments analysis and P-values of the molecular evolution test.
(4.05 MB XLS)

Table S2 Functional analysis of Prom+ genes, Cod+ genes and genes with positive selection either in the promoter or the coding region, showing the number of genes in the reference sets, the number of genes in the experimental sets and the p-values of a hypergeometric test for different PANTHER Ontology terms.
(0.05 MB PDF)

Table S3 Functional analysis of the set of genes with positive selection both in the promoter and in the protein-coding region, showing the number of genes in the reference set, the number of genes in the experimental set and the p-values of a hypergeometric test for different PANTHER Ontology terms.
(0.01 MB PDF)

Table S4 Data set of the centrality parameters.
(0.72 MB XLS)

Table S5 Main statistics of the distributions of the centrality parameters in Prom+ genes.
(0.01 MB PDF)

Table S6 Main statistics of the distributions of the centrality parameters in Cod+ genes.
(0.01 MB PDF)

Table S7 Level of expression for the positively selected genes.
(0.03 MB DOC)

Table S8 Functional analysis of the set of twenty genes containing the top ten central Prom+ genes and the top ten central Cod+ genes, showing the number of genes in the global reference set, the number of genes in the experimental set and the p-values of a hypergeometric test for different PANTHER Ontology terms.
(0.01 MB PDF)

Table S9 Functional analysis of the set of twenty genes containing the top ten central Prom+ genes and the top ten central Cod+ genes, showing the number of genes in the central reference set, the number of genes in the experimental set and the p-values of a hypergeometric test for different PANTHER Ontology terms.
(0.01 MB PDF)

Text S1 Potential effects of W->S bias on the centrality analysis.
(0.08 MB PDF)
