# Supplementary figures and images for: Gene Promoter Evolution Targets the Center of the Human Protein Interaction Network
Source: PLoS One. 2010 Jul 8;5(7):e11476. doi: 10.1371/journal.pone.0011476 (PMC2900212; doi:10.1371/journal.pone.0011476)

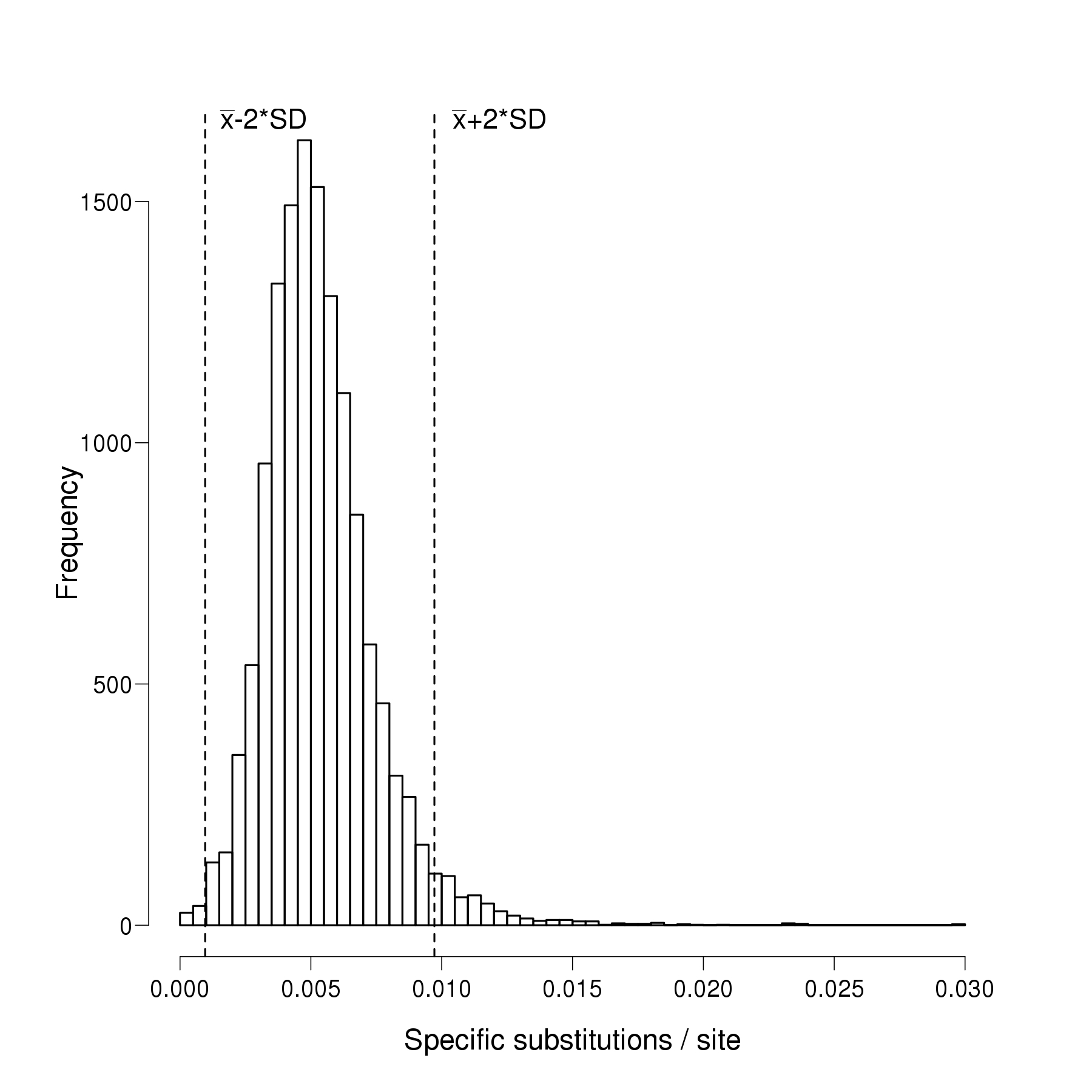

Supplement: Figure S1 — Distribution of the human specific substitutions per site in the intronic regions. The average human specific substitution ratio of the alignments of the intronic regions was 0.0053 and the standard deviation (s.d.) was 0.00219. (3.51 MB DOC) [file pone.0011476.s001.doc]
