# Supplementary material for: Gene Promoter Evolution Targets the Center of the Human Protein Interaction Network
Source: PLoS One. 2010 Jul 8;5(7):e11476. doi: 10.1371/journal.pone.0011476 (PMC2900212; doi:10.1371/journal.pone.0011476)
Supplement: Table S2 — Functional analysis of Prom+ genes, Cod+ genes and genes with positive selection either in the promoter or the coding region, showing the number of genes in the reference sets, the number of genes in the experimental sets and the p-values of a hypergeometric test for different PANTHER Ontology terms. (0.05 MB PDF) [file pone.0011476.s003.pdf]

Table S2. Functional analysis of *Prom*$^+$ genes, *Cod*$^+$ genes and genes with positive selection either in the promoter or the coding region (*Positive genes*), showing the number of genes in the reference sets, the number of genes in the experimental sets and the p-values of a hypergeometric test for different PANTHER Ontology terms.

**Biological Process**	**Reference *Prom*$^+$ genes**	***Prom*$^+$ genes**	**p-value**	**Reference *Cod*$^+$ genes**	***Cod*$^+$ genes**	**p-value**	**Reference *Positive genes***	***Positive genes***	**p-value**
Nucleoside, nucleotide and nucleic acid metabolism	928	67	0,186	789	64	0,336	1379	127	0,166
Signal transduction	878	54	0,015	820	66	0,305	1376	119	0,040
Protein metabolism	867	83	**0,040**	637	59	**0,272**	1195	140	**0,018**
Developmental processes	607	44	0,263	541	54	**0,123**	927	95	**0,397**
mRNA transcription	445	23	0,011	437	33	0,242	714	55	0,018
Protein modification	441	46	**0,035**	341	33	**0,251**	621	78	**0,017**
Cell surface receptor mediated signal transduction	354	30	**0,398**	369	24	0,080	606	54	0,206
Transport	417	31	0,370	285	19	0,140	562	50	0,214
Immunity and defense	371	26	0,269	293	18	0,073	535	43	0,069
Cell communication	336	13	0,001	301	26	**0,515**	508	39	0,041
Intracellular protein traffic	323	22	0,244	266	21	0,395	464	43	0,338
Cell structure and motility	290	25	**0,378**	255	22	**0,521**	450	46	**0,449**
Cell cycle	282	22	**0,585**	242	18	0,306	419	40	0,425
Intracellular signalling cascade	239	16	0,267	229	22	**0,316**	378	37	0,498
Cell proliferation and differentiation	227	18	**0,554**	230	19	0,492	372	37	**0,531**
Lipid, fatty acid and steroid metabolism	220	14	0,219	177	14	0,441	316	28	0,290
Other metabolism	188	22	**0,044**	133	12	**0,471**	252	32	**0,089**
Carbohydrate metabolism	157	16	**0,188**	151	18	**0,093**	249	34	**0,035**
Neuronal activities	151	8	0,135	150	18	**0,088**	245	26	**0,397**
Cell adhesion	161	10	0,247	133	15	**0,164**	240	24	**0,525**
Oncogenesis	132	6	0,087	127	11	**0,532**	204	17	0,258
Apoptosis	135	8	0,236	114	10	**0,518**	200	18	0,377
Protein biosynthesis	141	16	**0,097**	108	5	0,088	197	21	**0,405**
mRNA processing	96	7	0,493	81	7	**0,549**	138	14	**0,513**
Sensory perception	77	4	0,251	71	11	**0,038**	124	15	**0,251**
Amino acid metabolism	83	7	**0,501**	62	7	**0,279**	115	14	**0,253**
Protein targeting and localization	81	10	**0,111**	60	6	**0,410**	107	16	**0,064**
Electron transport	68	2	0,082	47	5	**0,377**	98	7	0,227
Homeostasis	68	4	0,356	47	1	0,079	94	5	0,083
Coenzyme and prosthetic group metabolism	67	8	**0,164**	37	4	**0,393**	86	12	**0,145**
Muscle contraction	64	3	0,235	39	8	**0,016**	81	11	**0,180**
Miscellaneous	48	5	**0,339**	40	4	**0,452**	69	9	**0,246**
Phosphate metabolism	51	7	**0,110**	23	5	**0,042**	56	12	**0,008**
Sulfur metabolism	22	3	**0,256**	27	2	**0,687**	43	5	**0,429**
Blood circulation and gas exchange	18	1	**0,778**	21	2	**0,548**	33	3	**0,652**
Nitrogen metabolism	3	1	**0,221**	6	0	1,000	8	1	**0,568**
Non-vertebrate process	5	0	1,000	3	0	1,000	6	0	1,000
