# Supplementary material for: Gene Promoter Evolution Targets the Center of the Human Protein Interaction Network
Source: PLoS One. 2010 Jul 8;5(7):e11476. doi: 10.1371/journal.pone.0011476 (PMC2900212; doi:10.1371/journal.pone.0011476)
Supplement: Table S3 — Functional analysis of the set of genes with positive selection both in the promoter and in the protein-coding region, showing the number of genes in the reference set, the number of genes in the experimental set and the p-values of a hypergeometric test for different PANTHER Ontology terms. (0.01 MB PDF) [file pone.0011476.s004.pdf]

Table S3. Functional analysis of the set of genes with positive selection both in the promoter and in the protein-coding region, showing the number of genes in the reference set, the number of genes in the experimental set and the p-values of a hypergeometric test for different PANTHER Ontology terms.

**Biological Process**	**Reference**	**Positive genes**	**p-value**
Cell proliferation and differentiation	95	7	0.01
Developmental processes	224	6	0.01
Nucleoside, nucleotide and nucleic acid metabolism	339	5	0.18
Signal transduction	327	4	0.35
Protein metabolism	312	4	0.32
Cell cycle	112	4	0.02
Cell structure and motility	96	2	0.22
Metabolism	69	2	0.13
mRNA processing	41	2	0.05
Cell adhesion	54	1	0.39
Immunity and defense	129	1	0.72
mRNA transcription	168	1	0.80
