# Supplementary material for: Gene Promoter Evolution Targets the Center of the Human Protein Interaction Network
Source: PLoS One. 2010 Jul 8;5(7):e11476. doi: 10.1371/journal.pone.0011476 (PMC2900212; doi:10.1371/journal.pone.0011476)
Supplement: Table S5 — Main statistics of the distributions of the centrality parameters in Prom+ genes. (0.01 MB PDF) [file pone.0011476.s006.pdf]

Table S5. Main statistics of the distributions of the centrality parameters in *Prom*$^+$ genes.

	***Prom*$^+$ genes**				***Prom*$^+$ genes reference**			
	Min.	Max.	Mean	Median	Min.	Max.	Mean	Median
**Degree**	1	162	7.42	3.00	1	348	6.57	3.00
**Betweenness**	0	0.017	0.00050	0.000032	0	0.049	0.00044	0.000016
**ASPL**	3.08	6.11	4.14	4.09	2.9	7.25	4.24	4.16
**EVC**	0.0000030	0.19	0.023	0.0059	0	1.00	0.019	0.0043

Prom$^+$ genes, n = 188. *Prom*$^+$ genes reference, n = 2219. Min.: the lowest value of the distribution. Max.: the highest value of the distribution.
