# Supplementary material for: Gene Promoter Evolution Targets the Center of the Human Protein Interaction Network
Source: PLoS One. 2010 Jul 8;5(7):e11476. doi: 10.1371/journal.pone.0011476 (PMC2900212; doi:10.1371/journal.pone.0011476)
Supplement: Table S6 — Main statistics of the distributions of the centrality parameters in Cod+ genes. (0.01 MB PDF) [file pone.0011476.s007.pdf]

Table S6. Main statistics of the distributions of the centrality parameters in *Cod*$^{+}$ genes.

	***Cod*$^{+}$ genes**				***Cod*$^{+}$ genes reference**			
	Min.	Max.	Mean	Median	Min.	Max.	Mean	Median
**Degree**	1	126	5.92	2.50	1	475	7.52	3.00
**Betweenness**	0	0.018	0.00040	0.000013	0	0.11	0.00053	0.000022
**ASPL**	3.27	5.82	4.29	4.26	2.79	8.02	4.22	4.15
**EVC**	0.0000070	0.22	0.010	0.0026	0	0.83	0.020	0.0047

Cod^{+} genes, n = 152. *Cod*$^{+}$ genes reference, n = 1811. min.: the lowest value of the distribution. max.: the highest value of the distribution.
