# Supplementary material for: Gene Promoter Evolution Targets the Center of the Human Protein Interaction Network
Source: PLoS One. 2010 Jul 8;5(7):e11476. doi: 10.1371/journal.pone.0011476 (PMC2900212; doi:10.1371/journal.pone.0011476)
Supplement: Table S7 — Level of expression for the positively selected genes. (0.03 MB DOC) [file pone.0011476.s008.doc]

**Table S7.** Level of expression for the positively selected genes.

|  |  | ***Prom^+^* genes** | ***Prom^+^* genes reference** | **p-value^3^** | ***Cod^+^* genes** | ***Cod^+^* genes reference** | **p-value^4^** | **p-value ^5^** |
| --- | --- | --- | --- | --- | --- | --- | --- | --- |
| **DATA1^1^** | **Mean** | 1069 | 899 |  | 823 | 920 |  |  |
|  | **Median** | 589 | 487 | 0.0801 | 390 | 510 | 0.0258 | 0.0121 |
| **DATA2^2^** | **Mean** | 605 | 563 |  | 516 | 570 |  |  |
|  | **Median** | 385 | 336 | 0.380 | 294 | 345 | 0.0295 | 0.0654 |

Expression data from GEO accession GSE803. *Prom^+^* genes, n = 132. *Prom^+^* genes reference, n = 1522. *Cod^+^* genes, n = 129. *Cod^+^* genes reference, n = 1522.

^1^ Using per gene the highest expression value encountered in the set of tissues included in the E-GEOD-803 experiment.

^2^ Using per gene the average of the expression values equal or greater than the median of the set of tissues included in the E-GEOD-803 experiment.

^3^ p-value corresponding to a one-tailed Wilcoxon-Mann-Whitney test comparing the distribution of the level of expression of *Prom^+^* genes and a reference distribution.

^4^ p-value corresponding to a one-tailed Wilcoxon-Mann-Whitney test comparing the distribution of the level of expression of *Cod^+^* genes and a reference distribution.

^5^ p-value corresponding to a one-tailed Wilcoxon-Mann-Whitney test comparing the distribution of the level of expression of *Prom^+^* genes and the distribution of the level of expression of *Cod^+^* genes.
