# Supplementary material for: Gene Promoter Evolution Targets the Center of the Human Protein Interaction Network
Source: PLoS One. 2010 Jul 8;5(7):e11476. doi: 10.1371/journal.pone.0011476 (PMC2900212; doi:10.1371/journal.pone.0011476)
Supplement: Table S8 — Functional analysis of the set of twenty genes containing the top ten central Prom+ genes and the top ten central Cod+ genes, showing the number of genes in the global reference set, the number of genes in the experimental set and the p-values of a hypergeometric test for different PANTHER Ontology terms. (0.01 MB PDF) [file pone.0011476.s009.pdf]

Table S8. Functional analysis of the set of twenty genes containing the top ten central *Prom*$^+$ genes and the top ten central *Cod*$^+$ genes (*Positive genes*), showing the number of genes in the global reference set, the number of genes in the experimental set and the p-values of a hypergeometric test for different PANTHER Ontology terms.

**Biological Process**	**Reference***	***Positive genes***	**p-value**
Signal transduction	1379	8	0.0098
Protein metabolism	1195	8	0.0040
Cell proliferation and differentiation	372	8	1.33e-06
Nucleoside, nucleotide and nucleic acid metabolism	1379	6	0.093
Cell cycle	419	5	0.0026
mRNA transcription	714	5	0.023
Intracellular protein traffic	464	4	0.021
Developmental processes	927	3	0.38
Cell structure and motility	450	2	0.29
Cell surface receptor mediated signal transduction	606	2	0.42
Apoptosis	200	2	0.080
Metabolism	252	2	0.12
mRNA processing	138	2	0.042
Phosphate metabolism	56	2	0.0075
Protein targeting and localization	107	2	0.026
Cell communication	508	1	0.71
Immunity and defense	535	1	0.73
Intracellular signalling cascade	378	1	0.60
Protein biosynthesis	197	1	0.38
Muscle contraction	81	1	0.18

*The reference set is obtained taking into account the union of the set of genes analyzed in this study and the set of genes analyzed in Berglund's work.
