# Supplementary material for: Gene Promoter Evolution Targets the Center of the Human Protein Interaction Network
Source: PLoS One. 2010 Jul 8;5(7):e11476. doi: 10.1371/journal.pone.0011476 (PMC2900212; doi:10.1371/journal.pone.0011476)
Supplement: Table S9 — Functional analysis of the set of twenty genes containing the top ten central Prom+ genes and the top ten central Cod+ genes, showing the number of genes in the central reference set, the number of genes in the experimental set and the p-values of a hypergeometric test for different PANTHER Ontology terms. (0.01 MB PDF) [file pone.0011476.s010.pdf]

Table S9. Functional analysis of the set of twenty genes containing the top ten central *Prom*$^+$ genes and the top ten central *Cod*$^+$ genes (*Positive genes*), showing the number of genes in the central reference set, the number of genes in the experimental set and the p-values of a hypergeometric test for different PANTHER Ontology terms.

**Biological Process**	**Reference***	***Positive genes***	**p-value**
Signal transduction	70	8	0.0048
Protein metabolism	164	8	0.40
Cell proliferation and differentiation	26	8	2.43e-06
Nucleoside, nucleotide and nucleic acid metabolism	101	6	0.25
Cell cycle	52	5	0.060
mRNA transcription	31	5	0.0071
Intracellular protein traffic	52	4	0.17
Developmental processes	27	3	0.10
Cell structure and motility	36	2	0.47
Cell surface receptor mediated signal transduction	22	2	0.24
Apoptosis	21	2	0.23
Metabolism	14	2	0.12
mRNA processing	23	2	0.26
Phosphate metabolism	8	2	0.042
Protein targeting and localization	12	2	0.089
Cell communication	12	1	0.41
Immunity and defense	30	1	0.74
Intracellular signalling cascade	28	1	0.72
Muscle contraction	6	1	0.23
Miscellaneous	4	1	0.16

*The reference set is obtained taking into account the union of the set of genes analyzed in this study and the set of genes analyzed in Berglund's work and selecting those having an Eigenvalue centrality (EVC) higher than the minimum EVC of positively selected central genes as defined.
