# Supplementary material for: Gene Promoter Evolution Targets the Center of the Human Protein Interaction Network
Source: PLoS One. 2010 Jul 8;5(7):e11476. doi: 10.1371/journal.pone.0011476 (PMC2900212; doi:10.1371/journal.pone.0011476)
Supplement: Text S1 — Potential effects of W->S bias on the centrality analysis. (0.08 MB PDF) [file pone.0011476.s011.pdf]

Text supplement for "Gene Promoter Evolution Targets the Center of the Human Protein Interaction Network" by Jordi Planas and Josep M. Serrat

Potential effects of W→S bias on the centrality analysis

This section presents a complementary analysis strengthening the confidence that positively selected promoters, unlike positively selected protein coding regions, belong to genes whose proteins are more central than expected in the protein interaction network. It has been observed that some sequences might have a biased pattern of AT to GC substitutions referred to as W→S bias (weak to strong)[1]. Though this phenomenon is likely to be caused by a fixation bias of these kinds of substitutions rather than a bias on mutation frequency, there may be some concerns that this bias is not the result of true positive selection. Since there is no way to assess this, we have developed a simple method to detect those genes that are more likely to be affected by this bias and we have removed them from the set of positively selected genes prior to applying the centrality analysis further.

We defined the bias of a genomic region as follows W→S bias = $(n_{W\rightarrow S}/AT)/((n_{W\rightarrow S}/AT)+(n_{S\rightarrow W}/GC))$, where $n_{W\rightarrow S}$ and $n_{S\rightarrow W}$ are the number of W→S and S→W substitutions respectively, and AT and GC are the AT and GC content of the ancestral sequence inferred by using parsimony. The data related to coding regions was obtained from Berglund *et al.*[1]

The distribution of the W→S bias in both coding regions and proximal promoters are distorted by large numbers of regions that either have a bias of zero or one due to the fact that either $n_{W\rightarrow S}$ and $n_{S\rightarrow W}$ are zero respectively (Fig. 1A and B). Since these values are a consequence of the short length of the sequences under analysis and the short evolutionary distance between human and chimp, and are not good estimates of the W→S bias, we simply removed them from further analysis yielding much more coherent distributions (Fig. 2B and C).

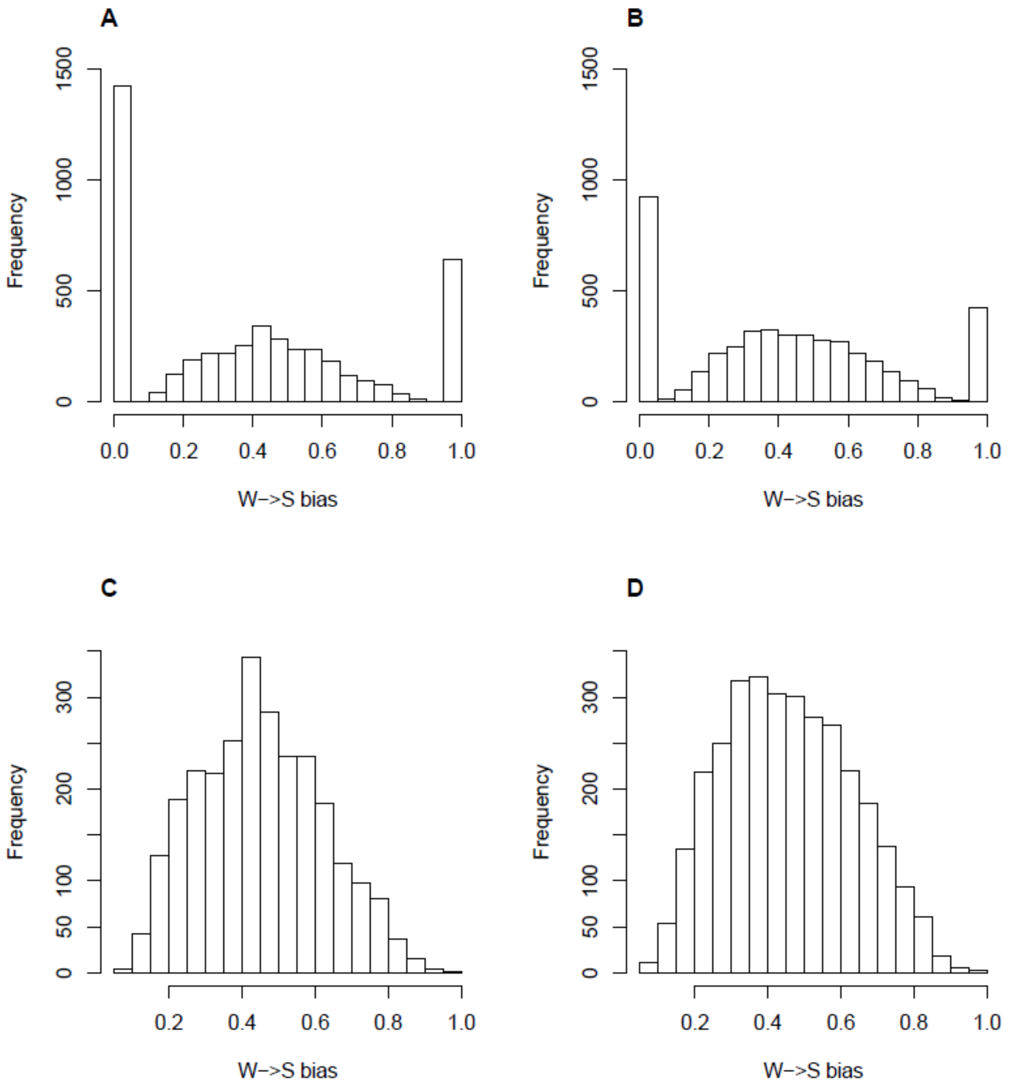

Figure 1. Distribution of W→S bias

We defined the genes being strongly influenced by the W→S bias as those having a bias greater or equal to the mean plus two times the standard deviation. Only genes with $P<0.05$ for the positive selection test were considered. In this category, we found 11 sequences in the Berglund's set of transcripts, 9 of which have an ENSEMBL gene code. In the promoters set, we found 10 sequences having a strong W→S bias. These genes were removed from the Cod^{+} and $Prom^{+}$ sets respectively (Table 1), and the centrality analysis was performed. Results show

that there are no qualitative changes with respect to the results presented in the main article, though the p-values of some of the analysis have experienced slight shifts (Table 2 and 3).

Table1. HGNC symbols of the genes showing a strong W→S

***Prom*⁺ genes**	***Cod*⁺ genes**
DOCK8	DMRT3
TEKT3	PIGS
ENOSF1	FAM178A
FBLN7	NUP153*
PIK3R1*	CANX*
MERTK	SLC4A10
LRRK1*	PWWP2B
SLC26A2	C20orf135
COLEC12*	MRGPRX2
PAIP1*	

* Genes coding for proteins present in the IntAct network

Table 2. Main statistics of the centrality distributions of *Prom*⁺ genes, excluding strong W→S genes

	***Prom*⁺ genes**					***Prom*⁺ reference**					**p-values**	
	n	*min.*	*max.*	*mean*	*median*	*n*	*min.*	*max.*	*mean*	*median*	*pv*[a]	*pv*[b]
**Degree**	186	1	162	7.5	3	2221	1	348	6.6	3	0.0084	*0.0094*
**Between.**	186	0	0.017	0.00050	0.000032	2221	0	0.049	0.00044	0.000016	0.0614	*0.071*
**ASPL**	186	3.077	6.11	4.14	4.091	2221	2.901	7.25	4.24	4.2	0.0045	*0.0044*
**EVC**	186	0.000003	0.19	0.023	0.0061	2221	0	1	0.019	0.0043	0.0082	*0.0080*

[a] One-tailed Wilcoxon-Mann-Whitney p-value
[b] One-tailed Wilcoxon-Mann-Whitney p-value before excluding strong W→S genes (p-values reported in the main text)

Table 3. Main statistics of the centrality distributions of *Cod*⁺ genes, excluding strong W→S genes

	***Cod*⁺ genes**					***Cod*⁺ reference**					**p-values**	
	n	*min.*	*max.*	*mean*	*median*	*n*	*min.*	*max.*	*mean*	*median*	*pv*[a]	*pv*[b]
**Degree**	150	1	126	5.9	2	1813	1	475	7.5	3	0.0283	*0.037*
**Between.**	150	0	0.018	0.00040	0.000013	1813	0	0.11	0.00053	0.000022	0.0675	*0.081*
**ASPL**	150	3.27	5.82	4.30	4.27	1813	2.8033	8.02	4.22	4.2	0.088	*0.014*
**EVC**	150	0.000007	0.22	0.010	0.0026	1813	0	0.83	0.020	0.0047	0.0037	*0.0063*

[a] One-tailed Wilcoxon-Mann-Whitney p-value
[b] One-tailed Wilcoxon-Mann-Whitney p-value before excluding strong W→S genes (p-values reported in the main text)

Although there is evidence that detection of positive selection in exons is influenced by a W→S bias, there are no conclusive results indicating that this effect is not related in someway to positive selection. We show that it does not affect the observation that positively selected

proteins are peripheral in the protein interaction network while proteins coded by genes with positively selected promoters are more central than expected.

References

1. Berglund J, Pollard KS, Webster MT. (2009) Hotspots of biased nucleotide substitutions in human genes. PLoS Biol 7(1): e26.
